# Spring Diseases

**Published:** 1859-04

**Authors:** 


### SPRING DISEASES.

A very large amount of the discomfort, lassitude, depression and actual sickness which prevails in the spring can be modified or wholly avoided by the exercise of a little reflection and self denial

One of the main objects of eating in winter time is to keep us warm, and to that end, provident nature gives a vigorous appetite in cold weather, but if as warm weather approaches we eat with the appetite of winter, the system not having time to adapt the appetite to the temperature, mischief will follow as inevitably as if a locomotive is diminished in speed one half while the fires are kept burning as fiercely as when it was moving at the utmost allowed rapidity. A prompt diminution at each meal of one-third of the amount eaten in winter, beginning before April in the latitude of New York, and earlier further south, would diminish spring diseases to an incalculable extent.

